# Growth Variation in the Pacific Oyster (*Crassostrea gigas*) Is Associated with Sequential Transcriptional Programs of Tissue Differentiation

**DOI:** 10.3390/ijms27198456

**Published:** 2026-09-22

**Authors:** Julan Kim, Hyojung Son, Hee-Jung Lee, Kyung-Hee Kim, Myunghee Jung, Dain Lee, Jaewon Lim, Hyejin Kim, Su Jin Park, Seung-won Seo, Hojin Jung, Ji-man Hong, Younhee Shin, Hee Jeong Kong

**Affiliations:** 1 Genetics and Breeding Research Center, National Institute of Fisheries Science (NIFS), Geoje 53334, Republic of Korea; kimjulan@korea.kr (J.K.);; 2 Research and Development Center, Insilicogen, Inc., Yongin 16954, Republic of Korea; 3 Southeast Sea Fisheries Research Institute, National Institute of Fisheries Science (NIFS), Tongyeong 39769, Republic of Korea

**Keywords:** crassostrea gigas, growth heterogeneity, developmental trajectory, tissue differentiation, transcriptome, biomineralization

## Abstract

Growth variation is one of the most economically important traits in oyster aquaculture, yet the transcriptional programs associated with divergent growth performance remain incompletely understood. In this study, three growth cohorts of the Pacific oyster (*Crassostrea gigas*) originating from the same production batch were examined to characterize coordinated differences in tissue-associated transcriptional programs across contrasting growth cohorts. A total of 25 RNA-seq libraries were generated from slow-growth (SG) whole-body samples and mantle and gill tissues of medium-growth (MG) and fast-growth (FG) oysters. RNA-seq analysis identified 28,773 expressed genes and 9373 differentially expressed genes (|log_2_FC| ≥ 2, FDR ≤ 0.05). Tissue-specific analysis further identified 1407 mantle-specific and 773 gill-specific genes, reflecting distinct functional specialization of the two tissues. Weighted gene co-expression network analysis (WGCNA) resolved six biologically interpretable modules associated with growth state and tissue identity. When module eigengene patterns were examined across the ordered SG–MG–FG continuum, the modules showed sequential shifts from SG-associated basal metabolic and RNA-processing programs to MG-associated tissue-specialization programs and FG-associated mature mantle and gill functional programs. The FG mantle-associated module was enriched for chitin remodeling, shell matrix-related functions, and structural organization, whereas the FG gill-associated module showed enrichment of Toll-like receptor signaling, epithelial organization, and innate immune functions. Expression patterns of representative genes from the six modules were independently validated by quantitative RT-PCR and showed strong concordance with the RNA-seq data. Collectively, these results identify coordinated differences in basal and tissue-associated transcriptional programs across contrasting growth cohorts. This framework provides a transcriptomic basis for interpreting growth-associated tissue maturation and identifies candidate molecular indicators for future validation in oyster selective breeding.

## 1. Introduction

The Pacific oyster, *Crassostrea gigas*, is one of the most economically important marine bivalves and has become a principal target species for selective breeding in modern aquaculture. Continued expansion of global oyster production has increased the demand for genetic improvement of commercially important traits, among which growth performance remains a primary breeding objective because shell size and body weight directly determine production efficiency, farming duration, and market value [1]. Recent advances in chromosome-level genome assemblies, high-density genetic markers, and genomic resources have greatly accelerated genetic studies and molecular breeding of oysters [2,3]. Nevertheless, oysters originating from the same production batch frequently exhibit substantial variation in growth, indicating that the molecular mechanisms underlying growth heterogeneity remain incompletely understood. Recent transcriptomic studies have identified numerous genes and signaling pathways associated with oyster growth, including oxidative phosphorylation, ribosome biogenesis, Wnt signaling, PI3K–Akt signaling, insulin-like peptide signaling, and energy metabolism [4,5,6,7]. These studies have substantially improved our understanding of the molecular basis of growth and have identified candidate genes for molecular breeding. However, most investigations have relied on pairwise comparisons between fast- and slow-growing individuals and have primarily interpreted growth-associated transcriptional variation in terms of differentially expressed genes and enriched pathways. Consequently, relatively little is known about how coordinated transcriptional programs associated with tissue specialization change across different growth states. Growth in bivalves depends on the coordinated development of tissues responsible for nutrient acquisition and shell formation. The mantle is the primary biomineralizing tissue that secretes shell matrix proteins and extracellular matrices required for shell formation [8], whereas the gill is essential for filter feeding, respiration, ion transport, and mucosal immune defense [9]. Previous tissue-specific transcriptomic studies have demonstrated distinct gene-expression programs associated with these specialized physiological functions [10]. However, growth-associated tissue specialization has remained difficult to investigate because extremely slow-growing oysters often fail to reach the size required for transfer to sea-cage culture and cannot be readily dissected into individual organs. These biological characteristics provide a unique opportunity to examine how transcriptional programs associated with tissue specialization differ among distinct growth states. In the present study, Pacific oysters originating from the same production batch were classified into slow-growth (SG), medium-growth (MG), and fast-growth (FG) cohorts according to realized growth performance. Bulk RNA sequencing was performed using whole-body samples from SG oysters and mantle and gill tissues from MG and FG oysters. Differential expression analysis, tissue-specific gene identification, weighted gene co-expression network analysis (WGCNA), functional enrichment analysis, and quantitative RT-PCR validation were subsequently integrated to characterize transcriptional programs associated with growth states and tissue specialization. By examining coordinated changes in co-expression modules across the SG–MG–FG continuum, this study provides a transcriptomic framework for understanding growth heterogeneity and identifies candidate molecular signatures that may facilitate future molecular breeding of *C. gigas*.

## 2. Results

### 2.1. Phenotypic Divergence Among Growth Cohorts

The three cohorts originated from the same production batch and were initially reared under identical land-based culture conditions. Once individuals reached the size required for sea transfer, the FG and MG cohorts were moved to the same sea-cage culture system and reared under identical environmental conditions until sampling. In contrast, SG individuals failed to attain the transfer size and therefore remained under land-based culture throughout the experimental period. At the time of sampling, the three cohorts exhibited striking phenotypic divergence (Figure 1). Mean shell height differed approximately 9.6-fold between SG (9.65 ± 1.11 mm) and FG (92.9 ± 5.99 mm), whereas mean total weight differed by more than two orders of magnitude (0.09 ± 0.02 g in SG versus 41.51 ± 6.87 g in FG) (Table 1). FG and MG oysters exhibited well-developed mantle lobes and clearly distinguishable gill tissues, enabling tissue-specific sampling, whereas SG individuals lacked sufficient anatomical differentiation for tissue dissection and were therefore analyzed as whole-body samples (Figure 1). Accordingly, 25 RNA-seq libraries were generated from five biological replicates for each sampling group, comprising five SG whole-body samples, five MG mantle samples, five MG gill samples, five FG mantle samples, and five FG gill samples (Table 1). This sampling design enabled comparative transcriptomic analyses of both growth-associated phenotypes and tissue-specific developmental programs.

### 2.2. Sequencing Performance and Tissue-Specific Transcriptional Divergence

Across the 25 RNA-seq libraries, a total of 153 Gb of paired-end sequencing data were generated, and all samples showed high RNA quality (RIN ≥ 8.9). The mean mapping rate to the *Crassostrea gigas* reference genome was 75.01% (Table S1). After expression filtering, 28,773 genes were retained for downstream analyses, representing the majority of annotated genes in the reference genome. Pairwise differential expression analysis identified 9373 DEGs (|log_2_FC| ≥ 2, FDR ≤ 0.05) across all group–tissue comparisons (Figure S1). The greatest transcriptional divergence was observed between the SG whole-body samples and the dissected tissues of MG and FG oysters, whereas numerous DEGs were also detected between mantle and gill tissues within the MG and FG groups. These results indicate that tissue identity contributed substantially to transcriptomic variation and suggest that tissue differentiation is a major component of growth-associated molecular divergence. To characterize these tissue-specific transcriptional programs, tissue-specific genes were identified using a tissue-specificity index (τ ≥ 0.75). A total of 1407 mantle-specific genes and 773 gill-specific genes were identified (Figure S2; Table S2). Functional enrichment analysis showed that mantle-specific genes were predominantly associated with biomineralization, shell matrix proteins (SMPs), chitin metabolism, and collagen organization, whereas gill-specific genes were enriched for ciliary motility, glycosaminoglycan biosynthesis, ion transport, and innate immune functions. These findings confirm that the transcriptional differences observed between tissues represent distinct functional differentiation programs rather than simple expression differences.

### 2.3. WGCNA Identifies Biologically Meaningful Transcriptional Modules Associated with Growth and Tissue Differentiation

To investigate the transcriptional programs underlying tissue differentiation during growth, weighted gene co-expression network analysis (WGCNA) was performed using the 9373 DEGs identified across the group–tissue comparisons. Based on co-expression patterns, eigengene profiles, and functional enrichment, six biologically meaningful modules were identified, representing distinct transcriptional programs associated with growth and tissue differentiation (Table S3). Functional enrichment analysis using Gene Ontology (GO) and Kyoto Encyclopedia of Genes and Genomes (KEGG) annotations demonstrated that each module was associated with a distinct set of biological processes and pathways (Table 2). Accordingly, the modules were designated according to their predominant biological characteristics as the SG basal metabolic (green), SG basal RNA-processing (yellow), mantle commitment (blue), gill commitment/ciliogenesis (turquoise), mantle terminal biomineralization (brown), and gill terminal immune maturation (red) modules.

### 2.4. Functional Characterization of Developmental Trajectory Modules

To determine how these six transcriptional programs were coordinated during growth, module eigengene expression profiles were integrated with the SG–MG–FG growth continuum and reinterpreted as a developmental pseudotime trajectory (Figure 2). Rather than representing simple group-specific expression patterns, the six modules exhibited sequential activation and repression along the reconstructed trajectory. To further characterize these developmental modules, their enriched biological functions, representative pathways, and hub genes were subsequently examined.

#### 2.4.1. SG Basal Metabolic Module (Green)—Minimal Energy Generation Without Tissue-Specific Demand

The SG Basal Metabolic Module exhibited its highest eigengene activity in SG whole-body samples and declined markedly along the subsequent developmental trajectory (Figure 2). Functional enrichment analysis identified oxidative phosphorylation, the tricarboxylic acid (TCA) cycle, central carbon metabolism, amino acid metabolism, and glutathione metabolism as major functional categories associated with this module (Table 2 and Table S3). Representative genes included mitochondrial glutathione transporter SLC25A40, V-type proton ATPase subunit D, acyl-coenzyme A thioesterase 13 (ACOT13), acyl-CoA-binding protein, an oxidoreductase YjmC homolog, and clathrin light chain A (Figure 3A). The functional composition of these genes is consistent with a transcriptional state dominated by fundamental metabolic and cellular homeostatic processes. SLC25A40 and the YjmC-like oxidoreductase are associated with mitochondrial/redox-related functions, whereas ACOT13 and acyl-CoA-binding protein link the module to acyl-CoA and lipid metabolism. V-type proton ATPase subunit D further reflects energy-dependent proton transport and intracellular homeostasis, while clathrin light chain A represents membrane trafficking accompanying basic cellular activity. Together with the enrichment of oxidative phosphorylation and TCA-cycle pathways, these patterns indicate that the Green module primarily represents a basal metabolic program supporting energy production, redox balance, and cellular maintenance in the SG state. Along the reconstructed developmental trajectory, Green-module activity decreased as mantle- and gill-associated modules became increasingly prominent (Figure 2). Its co-occurrence with the SG RNA-Processing Module in SG oysters therefore delineates an early transcriptional state characterized predominantly by general metabolic and gene-regulatory functions, prior to the emergence of tissue-associated transcriptional programs observed in MG and FG oysters.

#### 2.4.2. SG RNA Processing Module (Yellow)—Pre-Commitment Maintenance of Spliceosomal and mRNA-Surveillance Machinery

The SG RNA Processing Module showed its highest eigengene activity in SG whole-body samples, followed by a progressive decline along the reconstructed developmental trajectory (Figure 2). Functional enrichment analysis identified processes associated with RNA processing and regulation, including spliceosome-related functions, mRNA surveillance and decay, and RNA modification (Table 2 and Table S3). Representative genes included eukaryotic translation initiation factor 3 subunits M, B, and C (eIF3M, eIF3B, and eIF3C), mitochondrial import inner membrane translocase subunit TIM50, BOS complex subunit NCLN, and protein arginine N-methyltransferase 5 (PRMT5) (Figure 3B). The presence of multiple eIF3 subunits indicates that regulation of translation initiation constitutes an important component of this SG-associated module, while PRMT5 provides a functional link to RNA processing through its roles in post-transcriptional regulation and ribonucleoprotein maturation. TIM50 and NCLN further suggest that these regulatory processes are accompanied by fundamental mitochondrial and intracellular homeostatic functions. Together, the enrichment profile and representative gene composition indicate that the Yellow module reflects a basal gene-expression regulatory state involving coordinated RNA processing, translational control, and cellular maintenance, rather than a tissue-specific differentiation program. Along the developmental trajectory, Yellow-module activity declined as mantle- and gill-associated modules became increasingly prominent (Figure 2). This reciprocal pattern is consistent with a transition from the broad cellular regulatory program predominant in SG oysters toward more specialized transcriptional programs associated with mantle and gill differentiation.

#### 2.4.3. Mantle Commitment Module (Blue)—Wnt/ECM/PI3K Patterning Marks the Entry into Mantle Identity in MG

The Mantle Commitment Module exhibited a pronounced mantle-biased expression pattern, with increased eigengene activity accompanying the transition toward differentiated mantle tissue (Figure 2). Functional enrichment analysis identified Wnt signaling, ECM–receptor interaction, PI3K–Akt signaling, focal adhesion, and calcium signaling as major pathways associated with this module (Table 2 and Table S3). Representative genes included integrin alpha-4, integrin beta-3, fascin, filamin-A, plexin-B, and basement membrane-specific heparan sulfate proteoglycan core protein, which showed preferential expression in mantle tissues, particularly FG mantle, relative to gill tissues (Figure 3C). The functional composition of these representative genes supports a prominent role for cell–matrix interactions and cytoskeletal organization within the Blue module. Integrin alpha-4 and integrin beta-3 are membrane receptors involved in cell–ECM adhesion and associated signaling, whereas fascin and filamin-A contribute to actin cytoskeletal organization and cellular architecture. Basement membrane-specific heparan sulfate proteoglycan core protein provides an additional link to basement-membrane and extracellular-matrix organization, while plexin-B is consistent with signaling involved in cellular organization and tissue patterning. Together with the enrichment of ECM–receptor interaction and focal adhesion pathways, these expression patterns identify the Blue module as a mantle-associated developmental program characterized by coordinated remodeling of cell adhesion, extracellular matrix interactions, and cytoskeletal architecture. Along the reconstructed developmental trajectory, activation of the Blue module coincided with the decline of the SG-associated basal modules and preceded the stronger emergence of the Brown Mantle Terminal Biomineralization Module (Figure 2). This sequential pattern is consistent with an intermediate mantle differentiation state in which structural and cell–matrix organization precedes the later acquisition of specialized shell-forming functions.

#### 2.4.4. Gill Commitment/Ciliogenesis Module (Turquoise)—Motor-Protein and GAG Biosynthesis Program Defines MG Gill

The Gill Commitment/Ciliogenesis Module exhibited a pronounced gill-biased expression pattern, with increased activity during the intermediate developmental stage represented by MG gill (Figure 2). Functional enrichment analysis identified processes associated with ciliary and flagellar motility, axonemal organization, and glycosaminoglycan (GAG) biosynthesis (Table 2 and Table S3), consistent with the structural and functional development of the gill. Representative genes included axonemal dynein light chain domain-containing protein 1, dynein regulatory complex protein 10, doublecortin domain-containing protein 2, calcium/calmodulin-dependent protein kinase type IV (CaMKIV), nephrin, and STPG3, which showed preferential expression in gill tissues relative to mantle tissues (Figure 3D). Among these genes, axonemal dynein light chain domain-containing protein 1 and dynein regulatory complex protein 10 provide direct molecular support for the ciliogenesis-related annotation of this module, as both are components associated with the organization and regulation of axonemal motility. Doublecortin domain-containing protein 2, a microtubule-associated protein, further supports cytoskeletal organization accompanying the development of ciliated structures. The presence of CaMKIV, together with nephrin and STPG3, indicates that this structural program is accompanied by intracellular signaling and membrane-associated cellular organization. Collectively, these expression and enrichment patterns identify the Turquoise module as a gill-associated developmental program characterized primarily by the establishment and regulation of ciliary and cytoskeletal machinery. Along the reconstructed developmental trajectory, Turquoise-module activity became prominent during the transition from the SG state toward differentiated gill tissue and subsequently declined as the Red Gill Terminal Immune-Maturation Module increased (Figure 2). This pattern is consistent with an earlier stage of gill functional specialization in which establishment of the ciliary apparatus precedes the later immune-associated transcriptional state observed in FG gill.

#### 2.4.5. Mantle Terminal Biomineralization Module (Brown)—Acquisition of Mature Shell-Forming Functions

The Mantle Terminal Biomineralization Module exhibited a pronounced mantle-specific expression pattern, with eigengene activity increasing from MG to FG mantle while remaining low in gill tissues (Figure 2). Functional enrichment analysis identified the chitin catabolic process (GO:0006032), presynaptic membrane assembly (GO:0097105), and muscle structure development (GO:0061061) as major functional categories (Table 2 and Table S3). This pattern was supported by representative module genes, including serine-rich adhesin for platelets, temptin, asparagine-rich protein, ALX homeobox protein 1, follistatin-related protein 1, and neuroglobin, which were preferentially expressed in mantle tissues relative to the SG whole-body reference and showed markedly lower expression in gill tissues (Figure 3E). Among these genes, temptin, a component associated with the molluscan shell organic matrix, provides a direct link to shell-forming activity, while ALX homeobox protein 1 and follistatin-related protein 1 are consistent with developmental and tissue-remodeling processes. The mantle-biased expression of serine-rich adhesin for platelets and asparagine-rich protein further indicates changes in the extracellular environment accompanying mantle differentiation, whereas neuroglobin suggests concurrent changes in oxygen-related cellular physiology. Together with enrichment of chitin catabolism and muscle structure development, these gene-level patterns indicate that the Brown module integrates matrix remodeling, structural organization, and shell-associated functions during mantle maturation. Along the reconstructed developmental trajectory, increasing Brown-module activity from MG to FG mantle coincided with declining activity of the Mantle Commitment Module (Figure 2). This reciprocal pattern is consistent with a transcriptional transition from mantle establishment toward functional maturation and identifies the Brown module as a late-stage mantle program associated with acquisition of the shell-forming phenotype in FG oysters.

#### 2.4.6. Gill Terminal Immune-Maturation Module (Red)—Acquisition of Immune Competence in Mature Gill

The Gill Terminal Immune-Maturation Module exhibited increasing eigengene activity toward the late developmental trajectory, with the highest expression observed in FG gill (Figure 2). Functional enrichment analysis identified innate immune signaling, particularly Toll-like receptor signaling, together with processes associated with cell adhesion, cytoskeletal organization, and intracellular signal transduction (Table 2 and Table S3). Representative genes included Toll-like receptor 4 (TLR4), neural cell adhesion molecule 2 (NCAM2), Rho guanine nucleotide exchange factor TIAM1, Kelch-like protein 24 (KLHL24), zinc finger and BTB domain-containing protein 17 (ZBTB17), and polypyrimidine tract-binding protein 1 (PTBP1) (Figure 3F). Among these genes, TLR4 provides a direct link to innate immune recognition, whereas NCAM2 and TIAM1 are associated with cell adhesion and Rho-dependent cytoskeletal regulation, respectively. The presence of KLHL24 and ZBTB17, which are involved in protein homeostasis and transcriptional regulation, together with the RNA-binding protein PTBP1, further indicates coordinated regulation of cellular organization and gene expression within this module. Thus, the Red module appears to encompass not only immune-associated signaling but also cellular regulatory processes accompanying the acquisition of specialized gill functions. Along the reconstructed developmental trajectory, increasing Red-module activity coincided with declining activity of the Gill Commitment/Ciliogenesis Module (Figure 2), consistent with a shift from the earlier ciliogenesis-associated program toward a later transcriptional state characterized by enhanced immune and epithelial regulatory functions. Collectively, these patterns identify the Red module as a late-stage gill-associated transcriptional program linking innate immune signaling with the functional maturation of the gill in FG oysters.

### 2.5. Coordinated Developmental Reprogramming Links Tissue Differentiation to Growth

Taken together, the six developmental modules define a coordinated transcriptional cascade progressing from basal cellular maintenance (Green and Yellow), through tissue commitment (Blue and Turquoise), to terminal functional maturation (Brown and Red). Rather than functioning as independent gene sets, these modules were sequentially activated and replaced along the reconstructed developmental trajectory, indicating an ordered transition between distinct developmental states. Importantly, expression patterns of selected representative genes from each module were further examined by qRT-PCR and showed concordant trends with the corresponding RNA-seq data (Figure 4), supporting the reproducibility of these representative expression patterns. These findings support an association between growth variation in *Crassostrea gigas* and coordinated shifts in tissue-associated transcriptional programs rather than simple quantitative changes in individual genes. Collectively, these findings support the interpretation that differences in growth among SG, MG, and FG oysters are closely associated with the degree of developmental progression and tissue differentiation. This transcriptomic framework provides candidate molecular signatures linking organ maturation with somatic growth in *C. gigas*.

## 3. Discussion

### 3.1. Developmental Progression as a Molecular Basis of Growth Variation

Previous transcriptomic studies of growth variation in *C. gigas* have emphasized differential activity of energy metabolism, insulin-related signaling, and growth-associated pathways [4,5,6,7]. Our results are consistent with the involvement of these processes but extend this interpretation by showing that growth cohorts also differ in coordinated tissue-associated co-expression programs. By integrating WGCNA with a reconstructed developmental trajectory, we demonstrate that SG, MG, and FG represent sequential developmental states characterized by coordinated replacement of transcriptional programs rather than independent differential expression. This developmental framework provides a biological explanation for growth variation beyond simple differences in body size.

### 3.2. Functional Maturation of Mantle and Gill Underlies Rapid Growth

One of the major findings of this study is that terminal differentiation of the mantle and gill occurred in parallel with rapid growth. The mantle is the principal organ responsible for shell matrix secretion and biomineralization [8,10], whereas the gill functions as the primary interface for particle capture, respiration, and immune surveillance. Efficient shell deposition and sustained nutrient acquisition therefore require coordinated maturation of both organs [9,10]. The Brown module demonstrated that mantle maturation is accompanied by coordinated activation of chitin-remodeling enzymes, shell matrix proteins, neuroligin homologs, and extracellular matrix regulators. These genes likely contribute not only to shell formation itself but also to structural remodeling and neuroepithelial regulation required for sustained shell secretion. Likewise, the Red module showed that mature gills undergo extensive activation of Toll-like receptor signaling, epithelial organization, cytoskeletal remodeling, and innate immune pathways. Such coordinated activation is expected to enhance filtration efficiency while simultaneously protecting continuously exposed epithelial surfaces against environmental microorganisms. These observations suggest that accelerated oyster growth is achieved through synchronized maturation of organ-specific physiological functions rather than isolated activation of growth-related genes. In this context, developmental completion of the mantle and gill appears to represent a prerequisite for sustained somatic growth.

### 3.3. Implications for Transcriptome-Assisted Oyster Breeding

From a breeding perspective, the developmental framework proposed here provides an alternative strategy for identifying superior growth potential. Conventional breeding programs generally rely on phenotypic traits such as shell height or body weight measured after prolonged culture. However, our results indicate that oysters of the same chronological age may occupy different developmental states despite being reared under identical conditions. The sequential transcriptional modules identified in this study therefore represent candidate molecular indicators of growth-associated developmental status. In particular, genes associated with tissue specialization and functional maturation may provide complementary information to conventional morphological traits used in breeding programs. Nevertheless, their potential application as selection markers should be considered preliminary, and further validation across independent families, environments, and genetic backgrounds will be necessary before integration with genomic selection or marker-assisted breeding approaches [2,3].

### 3.4. Limitations and Future Perspectives

An important limitation of the present study is the unavoidable difference in sample composition among growth cohorts. Because SG oysters were too small for reliable anatomical separation of mantle and gill tissues, SG individuals were analyzed as whole-body samples, whereas MG and FG oysters were analyzed using dissected mantle and gill tissues. Consequently, transcriptional differences between SG and MG/FG may partly reflect differences in tissue composition in addition to biological variation associated with growth status. A second limitation is that SG oysters remained under land-based culture because they did not attain the size required for sea transfer, whereas MG and FG oysters were subsequently reared in sea cages. Thus, environmental differences between culture systems may also have contributed to the observed transcriptomic variation. Accordingly, the ordered SG–MG–FG expression patterns should be interpreted as a transcriptomic framework consistent with progressive tissue specialization, rather than direct evidence of a causal or temporally resolved developmental sequence. In addition, the trajectory was reconstructed from bulk RNA-seq data obtained from discrete growth states rather than from longitudinal sampling of the same individuals. Future studies using matched rearing environments, longitudinal sampling, and tissue-resolved or single-cell transcriptomic approaches will be required to disentangle growth-associated transcriptional changes from those attributable to tissue composition and environmental conditions. Finally, qRT-PCR validation was performed for selected representative genes rather than for all hub genes within each WGCNA module. Therefore, the qRT-PCR results provide independent support for representative expression trends but do not constitute experimental validation of the complete membership or network structure of the individual co-expression modules.

## 4. Materials and Methods

### 4.1. Experimental Animals, Rearing, and Cohort Definition

Pacific oyster (*C. gigas*) spat were produced at a hatchery in Geoje, Republic of Korea. After larval settlement, early spat were reared in land-based flow-through seawater tanks and fed Chaetoceros sp. (8 × 10^6^ cells/mL) twice daily. The tanks were maintained under a flow-through seawater system with complete water exchange twice daily. During nursery culture, spat were periodically size-graded according to growth as part of routine hatchery management to reduce size variation and competition among individuals. As the oysters grew, individuals reaching a suitable size for grow-out were transferred to a sea-based culture site in the Geoje coastal area, whereas the smallest individuals remained in the land-based hatchery because they had not attained sufficient size for sea transfer. No genetic modification or previous experimental procedures were involved. After seven months of culture, 12 growth groups were obtained through size-based selection. To minimize potential effects associated with cage location, groups were evenly distributed among the available cages. Individuals representing fast-, medium-, and slow-growth groups were identified based on shell height and total weight, and five individuals were randomly selected from each group for transcriptome analysis. No specific exclusion criteria were established. Three non-overlapping growth cohorts were defined based on shell height and total weight: fast-growth (FG; mean shell height 92.9 ± 5.99 mm, total weight 41.51 ± 6.87 g), medium-growth (MG; mean shell height 24.87 ± 2.73 mm, total weight 1.13 ± 0.51 g), and slow-growth (SG; mean shell height 9.65 ± 1.11 mm, total weight 0.09 ± 0.02 g). The FG and MG cohorts were sampled from the same sea-based grow-out site, whereas the SG cohort remained under land-based culture because of its small body size.

### 4.2. Tissue Sampling and Biological Replication

For FG and MG samples, gill and mantle were dissected under chilled sterile seawater, rinsed in diethyl pyrocarbonate-treated PBS, and flash-frozen in liquid nitrogen. For SG sample, tissues could not be resolved anatomically because the gill–mantle boundary, pallial cavity, and visceral mass had not reached the stage at which clean organ-level separation is feasible; whole-body samples were therefore collected and processed identically. Five independent biological replicates were obtained per group × tissue combination, yielding 25 tissue samples in total (FG gill, FG mantle, MG gill, MG mantle, SG whole body; *n* = 5 each). The sample size was determined based on the available biological samples and the experimental sampling design.

### 4.3. RNA Extraction, Library Preparation, and Sequencing

Total RNA was isolated with TRIzol reagent (Invitrogen, Carlsbad, CA, USA) followed by column purification (RNeasy Mini Kit, Qiagen, Hilden, Germany). Total RNA integrity was assessed using an Agilent 2100 Bioanalyzer or 4200 TapeStation (Agilent Technologies, Santa Clara, CA, USA); all 25 RNA samples exhibited RIN ≥ 8.9. Strand-specific mRNA libraries were constructed with the TruSeq Stranded mRNA Library Prep Kit (Illumina, San Diego, CA, USA) and sequenced on an Illumina NovaSeqX platform in 150 bp paired-end mode, generating approximately 153 Gb of raw data across the 25 libraries.

### 4.4. Read Quality Control, Alignment, and Quantification

Raw sequencing reads in FASTQ format were subjected to quality control procedures to remove adapter sequences, low-quality bases, and potential contaminants. Specifically, trimming and filtering were performed using Trimmomatic v0.39 [11] and BBDuk v38.26 [12] with default parameters unless otherwise specified. The resulting high-quality clean reads were aligned to the reference genome of Pacific oyster (*Crassostrea gigas*; assembly GCF_963853765.1_xbMagGiga1.1) using STAR v2.7.11a [13] with default settings optimized for paired-end RNA-seq data. Only uniquely mapped reads were retained for downstream analyses.

### 4.5. Expressed-Gene Calling and Differential Expression Analysis

Gene expression levels were quantified using RSEM v1.3.3, and expressed as transcripts per million (TPM) and raw read counts [14]. To correct for differences in library size among samples, normalization was performed using the trimmed mean of M-values (TMM) method implemented in edgeR v4.22 [15]. Expressed genes were defined as those with read count ≥ 5 and TPM ≥ 0.3 across all biological replicates within each growth group. Data quality and sample consistency were evaluated using boxplots, principal component analysis (PCA), Pearson correlation, and hierarchical clustering. Differential expression analysis was conducted using edgeR v4.22. Genes with |log_2_ fold change| ≥ 2 and false discovery rate (FDR) ≤ 0.05 were considered significantly differentially expressed genes (DEGs).

### 4.6. Identification of Tissue-Specific Genes

Tissue-specific genes were identified using the tissue-specificity index (τ), which quantifies the degree of preferential gene expression among biological groups [16]. To focus on tissue-specific transcription rather than growth-stage differences, MG and FG mantle samples were combined as the mantle group, and MG and FG gill samples were combined as the gill group, while SG whole-body samples were retained as a reference group. Genes with τ ≥ 0.75 and the highest mean TPM in either the mantle or gill group were classified as tissue-specific genes [17]. Functional enrichment analysis was performed using Gene Ontology (GO) [18,19] and Kyoto Encyclopedia of Genes and Genomes (KEGG) [20] annotations. Significantly enriched terms were identified using Fisher’s exact test with Benjamini–Hochberg correction [21] (FDR < 0.05).

### 4.7. Weighted Gene Co-Expression Network Analysis (WGCNA)

Weighted gene co-expression network analysis (WGCNA) was performed using the WGCNA (*Version: 1.74*) package in R to identify groups of co-expressed genes associated with growth state and tissue differentiation [22]. A signed co-expression network was constructed from variance-stabilized expression values of the 9373 differentially expressed genes (DEGs) across all 25 RNA-seq libraries. The soft-thresholding power was selected according to the scale-free topology criterion (R^2^ ≥ 0.85). Gene modules were identified by hierarchical clustering based on the topological overlap matrix (TOM) using the dynamic tree-cut algorithm with a minimum module size of 30 genes [23], followed by module merging at an eigengene dissimilarity threshold of 0.25 [19]. Module eigengenes, representing the first principal component of each module, were calculated to summarize module expression profiles and compared among the SG, MG, and FG sample groups. Representative modules showing distinct eigengene expression patterns were subsequently subjected to Gene Ontology (GO) and Kyoto Encyclopedia of Genes and Genomes (KEGG) enrichment analyses. Based on their eigengene profiles together with significantly enriched GO terms and KEGG pathways, six biologically meaningful modules were selected for downstream interpretation. Descriptive module names were assigned according to the predominant biological processes represented by each module.

### 4.8. Quantitative Real-Time PCR Validation

To independently assess representative RNA-seq expression patterns, genes were selected from each co-expression module by jointly considering high intramodular connectivity and representation of the major significantly enriched GO/KEGG functional categories, rather than absolute expression level alone. Total RNA was reverse-transcribed into first-strand cDNA using Transcriptor First Strand cDNA Synthesis Kit (Roche, Basel, Switzerland) according to the manufacturer’s instructions. Quantitative real-time PCR (qRT-PCR) was performed using Fast SYBR Green Master Mix (Applied Biosystems, Inc., Foster City, CA, USA) on a QuantStudio 5 Real-Time PCR System (Applied Biosystems, Inc.) following the manufacturer’s recommended cycling conditions. Relative gene expression levels were calculated using the 2^−ΔΔCt^ method [24] with RS18 as the internal control, where ΔCt = Ct(target) − Ct(RS18) and ΔΔCt = ΔCt(sample) − mean ΔCt(SG whole-body group). Five biological replicates were analyzed for each group, and each reaction was performed in technical triplicate; technical-replicate Ct values were averaged before ΔCt calculation. Relative expression was expressed as log2 fold change relative to the SG whole-body group and compared, on the same scale, with the RNA-seq fold changes (edgeR log2 fold change, Section 4.5). Results are presented as mean ± standard deviation (n = 5). Statistical significance was assessed on ΔCt values by one-way analysis of variance (ANOVA) followed by Dunnett’s multiple comparison test against the SG whole-body group (* *p* < 0.05, ** *p* < 0.01, *** *p* < 0.001). The concordance between RNA-seq and qRT-PCR expression patterns was assessed to evaluate the reproducibility of the selected transcriptomic expression trends. Statistical analyses were performed in R v4.4.1 using the *multcomp* (v1.4-29) and *stats* (v4.5.2) packages, and figures were generated using *ggplot2*.(v4.0.3) Primer sequences used in this study are listed in Table S4.

## 5. Conclusions

This study identifies coordinated shifts in transcriptional programs across slow-, medium-, and fast-growth Pacific oysters, progressing from SG-associated basal cellular functions to tissue-specific commitment and mature mantle and gill functions. The concordant WGCNA, functional enrichment, tissue-specific expression, and qRT-PCR results support an association between growth heterogeneity and the extent of tissue differentiation and functional maturation. These developmental module signatures provide candidate molecular indicators for future validation in studies of oyster growth and selective breeding.

## Figures and Tables

**Figure 1:**
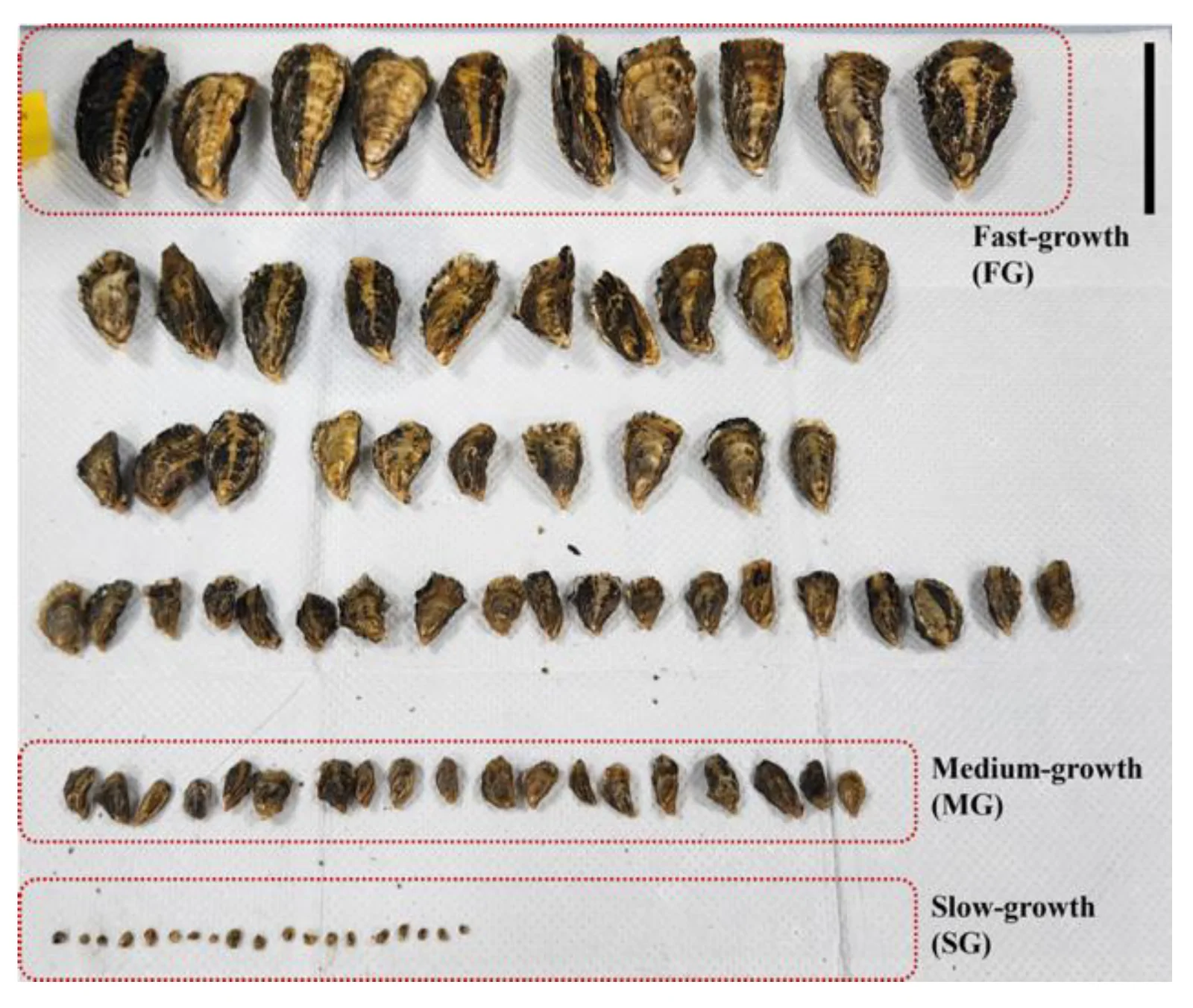
Classification of Pacific oyster growth phenotypes used in this study. Representative individuals from the fast-growth (FG), medium-growth (MG), and slow-growth (SG) groups are shown. Oysters were classified according to growth performance based on morphometric measurements and subsequently used for tissue-specific transcriptome analysis. The FG, MG, and SG groups represent distinct growth states that were used to investigate growth-associated gene expression patterns, tissue differentiation, and developmental progression. Scale bar, 10 cm.

**Figure 2:**
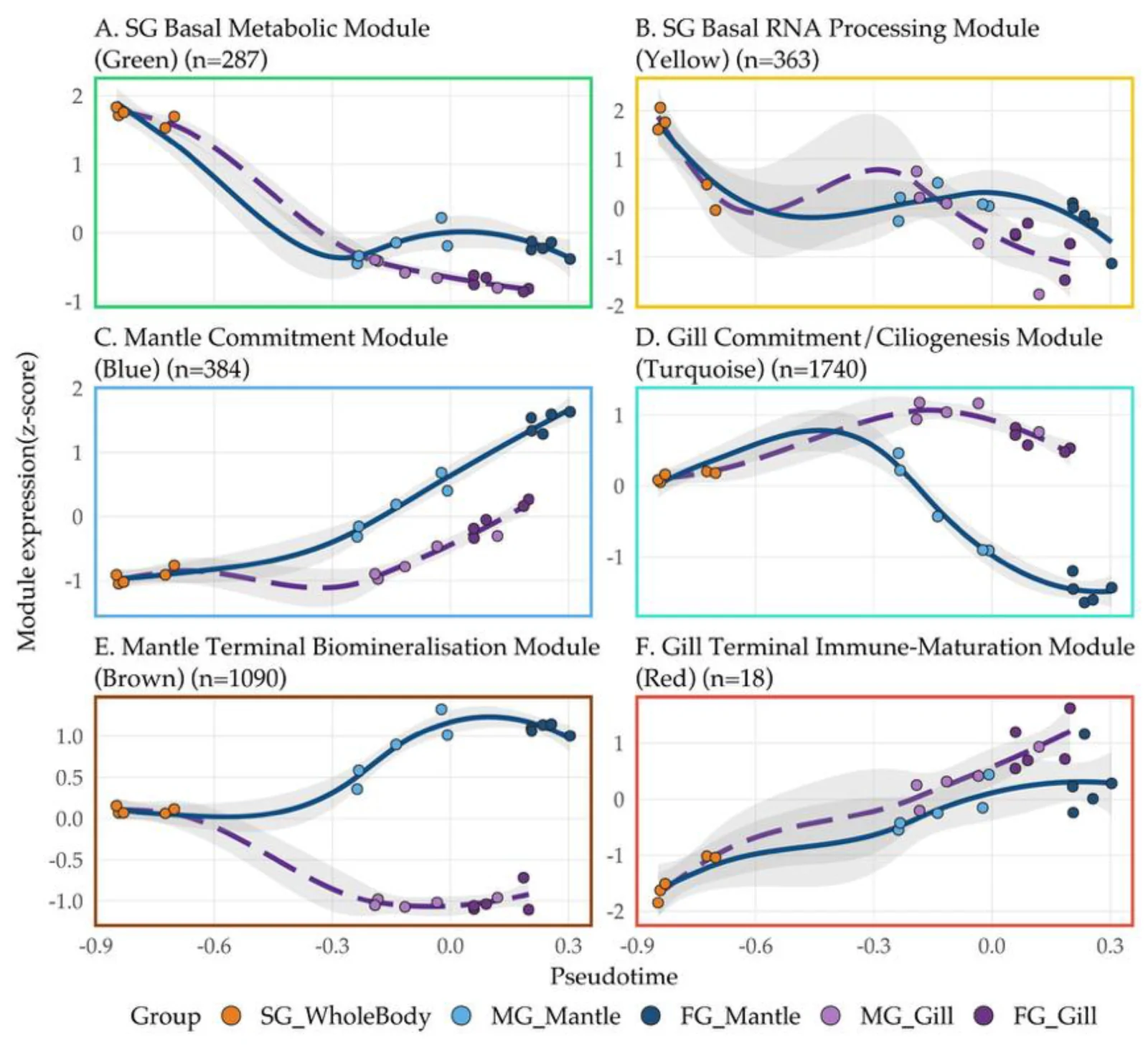
WGCNA module eigengene patterns across the ordered SG–MG–FG continuum. Eigengene expression profiles are shown for the six functionally annotated co-expression modules. Points represent individual samples from SG whole body, MG mantle, FG mantle, MG gill, and FG gill groups, as indicated by the legend. Module sizes are shown in parentheses. The horizontal axis represents a conceptual ordering of the SG–MG–FG growth states and does not represent directly measured developmental time. Solid and dashed lines denote mantle and gill trajectories, respectively, while shaded areas indicate the variability in gene expression around the fitted median trend.

**Figure 3:**
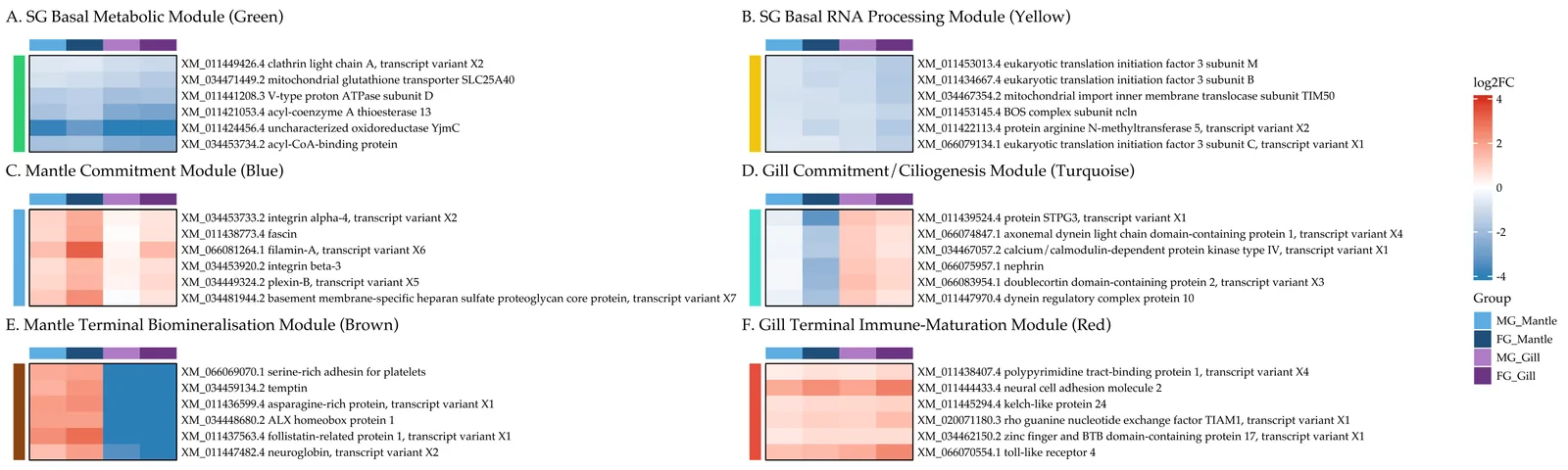
Expression patterns of representative genes from the six functionally annotated WGCNA modules. (**A**) SG basal metabolic, (**B**) SG basal RNA-processing, (**C**) mantle commitment, (**D**) mantle terminal biomineralization, (**E**) gill commitment/ciliogenesis, and (**F**) gill terminal immune-maturation modules. Representative genes were selected based on module connectivity and their association with the predominant enriched functional categories. Expression values are shown relative to SG whole-body samples. Columns represent MG mantle, FG mantle, MG gill, and FG gill, respectively; red and blue indicate relative up- and downregulation.

**Figure 4:**
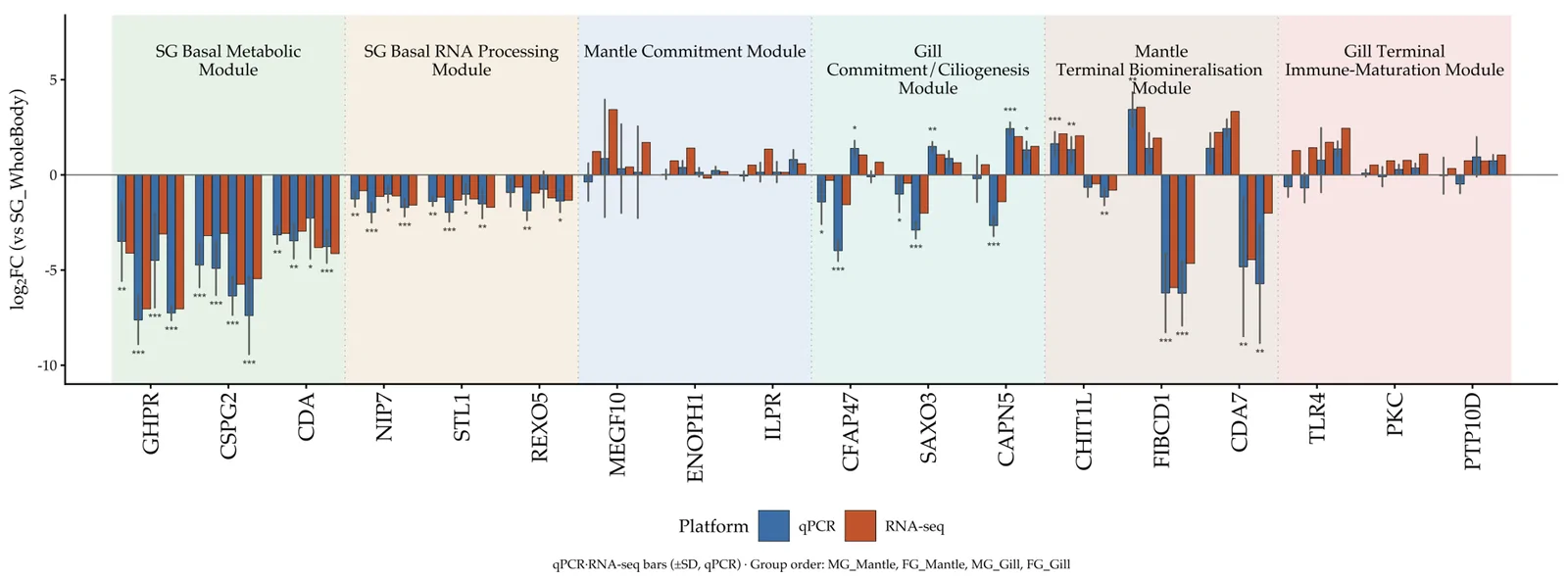
Validation of representative developmental module genes by quantitative real-time PCR (qRT-PCR). Representative genes selected from each of the six developmental co-expression modules were validated by quantitative real-time PCR (qRT-PCR). Relative expression levels are presented as log_2_ fold change compared with the SG whole-body group and are shown alongside the corresponding RNA-seq results. Genes are organized according to the developmental modules identified by WGCNA, illustrating the sequential transition from basal metabolic and RNA-processing programs (SG), through tissue commitment (MG), to terminal functional differentiation (FG). The concordant expression trends observed between qRT-PCR and RNA-seq for the selected representative genes support the reproducibility of the corresponding RNA-seq expression patterns. Blue bars indicate qRT-PCR expression levels, and orange bars indicate RNA-seq expression levels. Error bars represent standard deviation of qRT-PCR biological replicates. Statistical significance relative to the SG whole-body group is indicated as *p* < 0.05. The number of asterisks indicates the level of statistical significance: * *p* < 0.05, ** *p* < 0.01, and *** *p* < 0.001.

**Table 1:** Phenotypic and sampling design across cohorts. FG, MG, and SG oysters were classified according to shell height and total weight. Gill and mantle tissues were sampled from FG and MG individuals, while whole-body samples were collected from SG individuals. Five biological replicates were analyzed for each tissue or cohort.

Cohort	Shell Height (mm, Mean)	Total Weight (g, Mean)	Tissues Sampled	Biological Replicates
FG	92.90 ± 5.99	41.51 ± 6.87	Gill, Mantle	5 per tissue
MG	24.87 ± 2.73	1.13 ± 0.51	Gill, Mantle	5 per tissue
SG	9.65 ± 1.11	0.09 ± 0.02	Whole body	5

**Table 2:** Functional annotation of WGCNA modules across the reconstructed developmental trajectory. Modules were assigned to sequential pseudotime states based on eigengene expression patterns observed across SG, MG, and FG cohorts. Peak expression groups and representative enriched pathways or Gene Ontology (GO) terms are presented for each module.

Pseudotime Stage	Module (Colour)	Peak Group × Tissue	Core Pathway/Ontology Footprint	*p*_Value
Pre-commitment (SG)	SG Basal Metabolic Module (Green)	SG whole body	Crg00190 Oxidative Phosphorylation	1.11 × 10^−27^
			Crg00020 Citrate Cycle (TCA Cycle)	9.41 × 10^−8^
	SG Basal RNA Processing Module (Yellow)	SG whole body	Crg03008 Ribosome Biogenesis	8.62 × 10^−25^
			Crg00970 Aminoacyl-tRNA Biosynthesis	9.19 × 10^−11^
			Crg04141 Protein Processing In Endoplasmic Reticulum	5.14 × 10^−10^
Tissue commitment (MG)	Mantle Commitment (Blue)	MG mantle	Crg04820 Cytoskeleton In Muscle Cells	2.04 × 10^−23^
			Crg04512 ECM–Receptor Interaction	7.36 × 10^−8^
			Crg04310 Wnt Signaling	7.60 × 10^−6^
	Gill Commitment/Ciliogenesis (Turquoise)	MG gill	Crg04080 Neuroactive Ligand–Receptor Interaction	2.07 × 10^−7^
			Crg00534 Glycosaminoglycan Biosynthesis	9.18 × 10^−6^
			GO:0060294 Cilium Movement Involved In Cell Motility	7.64 × 10^−22^
Terminal differentiation (FG)	Mantle Terminal Biomineralisation (Brown)	FG mantle	GO:0097105 Presynaptic Membrane Assembly	1.58 × 10^−6^
			GO:0006032 Chitin Catabolic Process	2.87 × 10^−6^
			GO:0061061 Muscle Structure Development	5.42 × 10^−4^
	Gill Terminal Immune-Maturation (Red)	FG gill	Crg04620 Toll-Like Receptor Signaling Pathway	3.75 × 10^−3^
			Crg04517 IgSF CAM Signaling	7.97 × 10^−4^
			GO:0006955 Immune Response	3.39 × 10^−4^

## Data Availability

The raw RNA sequencing datasets generated during this study have been deposited in the NCBI Sequence Read Archive (SRA) under BioProject accession number PRJNA1515498. The processed tissue-specific gene lists and functional enrichment results supporting the findings of this study are provided in Supplementary Materials.

## Supplementary Materials

The following supporting information can be downloaded at: https://www.mdpi.com/article/10.3390/ijms27198456/s1.

## Abbreviations

The following abbreviations are used in this manuscript:

FG	Fast-Growth
MG	Medium-Growth
SG	Slow-Growth
GO	Gene Ontology
WGCNA	Weighted gene co-expression network analysis
