# Supplementary material for: Growth Variation in the Pacific Oyster (Crassostrea gigas) Is Associated with Sequential Transcriptional Programs of Tissue Differentiation
Source: Int J Mol Sci. 2026 Sep 22;27(19):8456. doi: 10.3390/ijms27198456 (PMC13664851; doi:10.3390/ijms27198456)
Supplement: Supplementary file 1 [file ijms-27-08456-s001.zip › Figure S1.pdf]

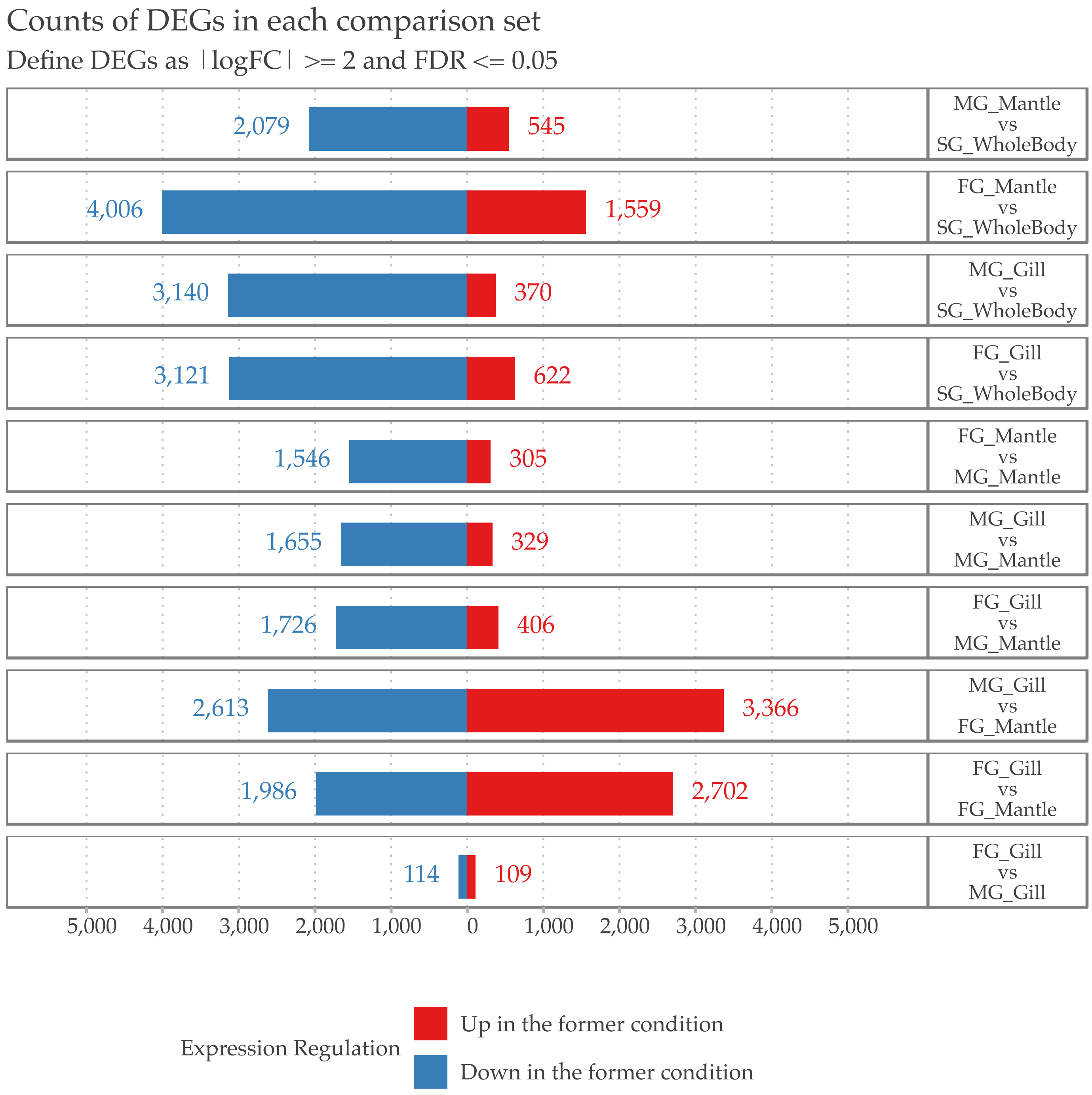
Counts of DEGs in each comparison set
Define DEGs as |logFC| >= 2 and FDR <= 0.05
2,079
545
MG_Mantle vs SG_WholeBody
4,006
1,559
FG_Mantle vs SG_WholeBody
3,140
370
MG_Gill vs SG_WholeBody
3,121
622
FG_Gill vs SG_WholeBody
1,546
305
FG_Mantle vs MG_Mantle
1,655
329
MG_Gill vs MG_Mantle
1,726
406
FG_Gill vs MG_Mantle
2,613
3,366
MG_Gill vs FG_Mantle
1,986
2,702
FG_Gill vs FG_Mantle
114
109
FG_Gill vs MG_Gill
5,000 4,000 3,000 2,000 1,000 0 1,000 2,000 3,000 4,000 5,000
Expression Regulation
Up in the former condition
Down in the former condition
