# Supplementary material for: Growth Variation in the Pacific Oyster (Crassostrea gigas) Is Associated with Sequential Transcriptional Programs of Tissue Differentiation
Source: Int J Mol Sci. 2026 Sep 22;27(19):8456. doi: 10.3390/ijms27198456 (PMC13664851; doi:10.3390/ijms27198456)
Supplement: Supplementary file 1 [file ijms-27-08456-s001.zip › Figure S2.pdf]

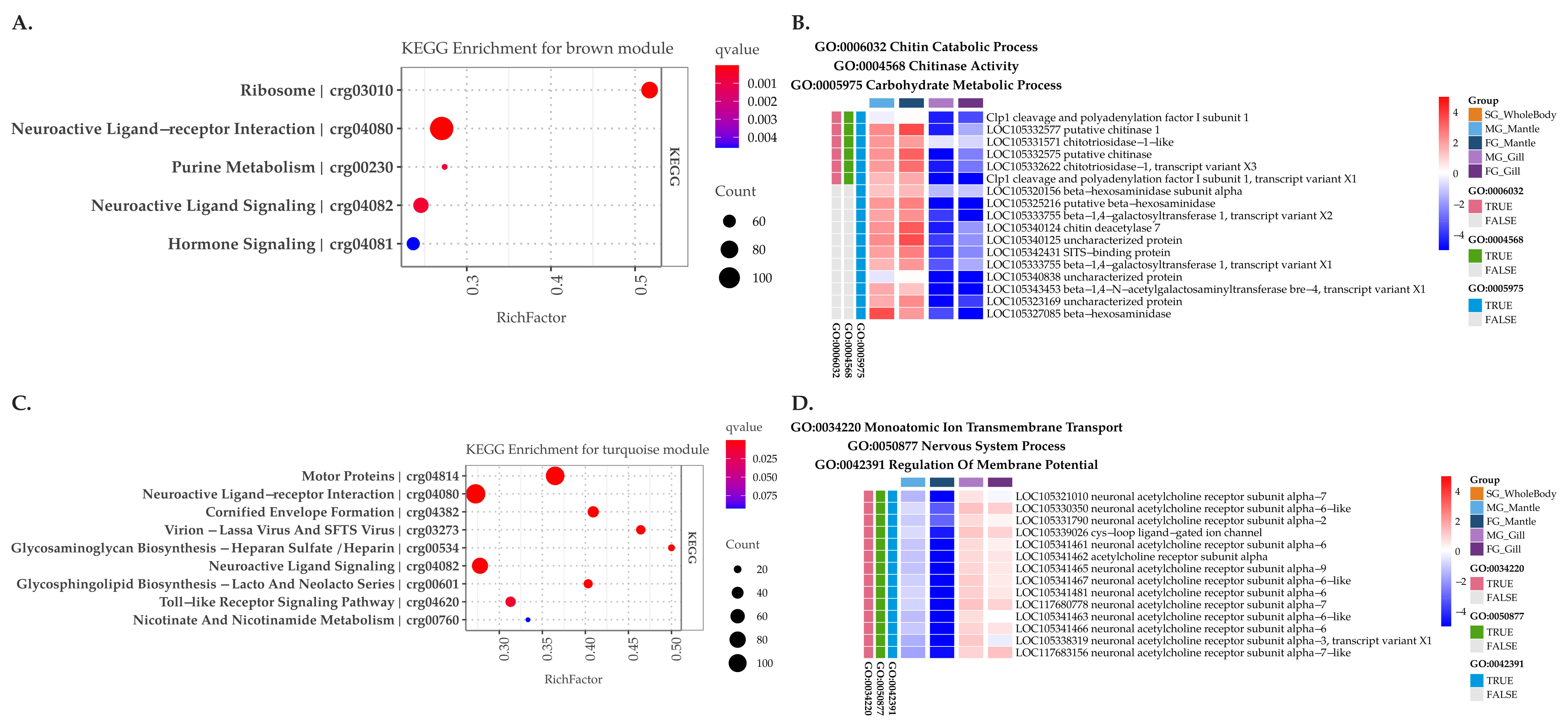
A.
KEGG Enrichment for brown module
Ribosome | crg03010
Neuroactive Ligand−receptor Interaction | crg04080
Purine Metabolism | crg00230
Neuroactive Ligand Signaling | crg04082
Hormone Signaling | crg04081
0.3
0.4
0.5
RichFactor
KEGG
qvalue
0.001
0.002
0.003
0.004
Count
60
80
100
B.
GO:0006032 Chitin Catabolic Process
GO:0004568 Chitinase Activity
GO:0005975 Carbohydrate Metabolic Process
Clp1 cleavage and polyadenylation factor I subunit 1
LOC105332577 putative chitinase 1
LOC105331571 chitotriosidase−1−like
LOC105332575 putative chitinase
LOC105332622 chitotriosidase−1, transcript variant X3
Clp1 cleavage and polyadenylation factor I subunit 1, transcript variant X1
LOC105320156 beta−hexosaminidase subunit alpha
LOC105325216 putative beta−hexosaminidase
LOC105333755 beta−1,4−galactosyltransferase 1, transcript variant X2
LOC105340124 chitin deacetylase 7
LOC105340125 uncharacterized protein
LOC105342431 SITS−binding protein
LOC105333755 beta−1,4−galactosyltransferase 1, transcript variant X1
LOC105340838 uncharacterized protein
LOC105343453 beta−1,4−N−acetylgalactosaminyltransferase bre−4, transcript variant X1
LOC105323169 uncharacterized protein
LOC105327085 beta−hexosaminidase
GO:0006032
GO:0004568
GO:0005975
4
2
0
−2
−4
Group
SG_WholeBody
MG_Mantle
FG_Mantle
MG_Gill
FG_Gill
GO:0006032
TRUE
FALSE
GO:0004568
TRUE
FALSE
GO:0005975
TRUE
FALSE
C.
KEGG Enrichment for turquoise module
Motor Proteins | crg04814
Neuroactive Ligand−receptor Interaction | crg04080
Cornified Envelope Formation | crg04382
Virion − Lassa Virus And SFTS Virus | crg03273
Glycosaminoglycan Biosynthesis − Heparan Sulfate / Heparin | crg00534
Neuroactive Ligand Signaling | crg04082
Glycosphingolipid Biosynthesis − Lacto And Neolacto Series | crg00601
Toll−like Receptor Signaling Pathway | crg04620
Nicotinate And Nicotinamide Metabolism | crg00760
0.30
0.35
0.40
0.45
0.50
RichFactor
KEGG
qvalue
0.025
0.050
0.075
Count
20
40
60
80
100
D.
GO:0034220 Monoatomic Ion Transmembrane Transport
GO:0050877 Nervous System Process
GO:0042391 Regulation Of Membrane Potential
LOC105321010 neuronal acetylcholine receptor subunit alpha−7
LOC105330350 neuronal acetylcholine receptor subunit alpha−6−like
LOC105331790 neuronal acetylcholine receptor subunit alpha−2
LOC105339026 cys−loop ligand−gated ion channel
LOC105341461 neuronal acetylcholine receptor subunit alpha−6
LOC105341462 acetylcholine receptor subunit alpha
LOC105341465 neuronal acetylcholine receptor subunit alpha−9
LOC105341467 neuronal acetylcholine receptor subunit alpha−6−like
LOC105341481 neuronal acetylcholine receptor subunit alpha−6
LOC117680778 neuronal acetylcholine receptor subunit alpha−7
LOC105341463 neuronal acetylcholine receptor subunit alpha−6−like
LOC105341466 neuronal acetylcholine receptor subunit alpha−6
LOC105338319 neuronal acetylcholine receptor subunit alpha−3, transcript variant X1
LOC117683156 neuronal acetylcholine receptor subunit alpha−7−like
GO:0034220
GO:0050877
GO:0042391
4
2
0
−2
−4
Group
SG_WholeBody
MG_Mantle
FG_Mantle
MG_Gill
FG_Gill
GO:0034220
TRUE
FALSE
GO:0050877
TRUE
FALSE
GO:0042391
TRUE
FALSE
