# Supplementary material for: Growth Variation in the Pacific Oyster (Crassostrea gigas) Is Associated with Sequential Transcriptional Programs of Tissue Differentiation
Source: Int J Mol Sci. 2026 Sep 22;27(19):8456. doi: 10.3390/ijms27198456 (PMC13664851; doi:10.3390/ijms27198456)
Supplement: Supplementary file 1 [file ijms-27-08456-s001.zip › Supplementary Figure and Table Legends.pdf]

Supplementary Materials

Figure S1. Differentially expressed genes across pairwise comparisons of growth cohorts and tissues. Bar plots show the numbers of differentially expressed genes (DEGs) identified in pairwise comparisons among SG whole-body, MG mantle, FG mantle, MG gill, and FG gill samples. DEGs were defined using thresholds of $|\log_2$ fold change $(\log_2 FC)| \geq 2$ and false discovery rate (FDR) ≤ 0.05. For each comparison, red bars indicate genes upregulated in the former condition relative to the latter condition, whereas blue bars indicate genes downregulated in the former condition. Comparisons include MG mantle vs. SG whole body, FG mantle vs. SG whole body, MG gill vs. SG whole body, FG gill vs. SG whole body, FG mantle vs. MG mantle, MG gill vs. MG mantle, FG gill vs. MG mantle, MG gill vs. FG mantle, FG gill vs. FG mantle, and FG gill vs. MG gill. Numbers adjacent to the bars indicate the number of DEGs in each direction.

Figure S2. Expression patterns and functional characteristics of tissue-specific genes identified in mantle and gill tissues of *Crassostrea gigas*. Tissue-specific genes were defined using a tissue-specificity index ($\tau \geq 0.8$). Heatmaps show representative mantle-specific and gill-specific genes across growth groups and tissues. Accompanying enrichment analyses summarize the major biological processes and pathways associated with each tissue-specific gene set. Mantle-specific genes were predominantly enriched for biomineralization-related functions, including chitin metabolism, shell matrix formation, and extracellular matrix organization, whereas gill-specific genes were enriched for neural signaling, membrane transport, ciliogenesis, and immune-related processes.

Table S1. **RNA quality control, sequencing output, and read mapping statistics of samples used for transcriptome analysis.** For each biological replicate, RNA concentration, total RNA amount, RNA integrity number (RIN), sequencing yield, percentage of retained reads after preprocessing, and percentage of reads mapped to the *Crassostrea gigas* reference genome are reported.

Table S2. List of tissue-specific genes identified in mantle and gill tissues. Tissue-specific genes were defined using a tissue-specificity index ($\tau \geq 0.8$). The table provides gene identifiers, functional annotations, tissue-specificity classification, and associated expression metrics used for downstream analyses.

Table S3. Enriched GO and KEGG functional categories and corresponding gene members for the six developmental trajectory clusters. Trajectory-associated genes were grouped into six clusters according to their expression dynamics along the reconstructed pseudotime axis. Functional enrichment analysis was performed using Fisher's exact test, and significantly enriched GO terms and KEGG pathways were identified for each cluster. For each enriched category, the corresponding gene members, functional annotations, enrichment statistics, and significance values are provided.

Table S4. Primer sequences used for quantitative real-time PCR (qRT-PCR) validation. Primer sequences used for qRT-PCR validation of representative genes selected from the six developmental co-expression modules identified by weighted gene co-expression network analysis (WGCNA). Transcript IDs, forward and reverse primer sequences, and expected amplicon sizes are provided.
